# Left ventricular strain patterns and their relationships with cardiac biomarkers in hypertrophic cardiomyopathy patients with preserved left ventricular ejection fraction

**DOI:** 10.3389/fcvm.2022.963110

**Published:** 2022-10-04

**Authors:** Lisha Zhang, Yixuan Wan, Bo He, Lei Wang, Dongyong Zhu, Fabao Gao

**Affiliations:** ^1^Department of Radiology, West China Hospital, Sichuan University, Chengdu, China; ^2^Molecular Imaging Center, West China Hospital, Sichuan University, Chengdu, China

**Keywords:** hypertrophic cardiomyopathy, cardiac magnetic resonance feature tracking, cardiac troponin T, N-terminal prohormone of the brain natriuretic peptide, strain

## Abstract

**Aims:**

This study aims to assess left ventricular (LV) function in hypertrophic cardiomyopathy (HCM) patients with preserved left ventricular ejection fraction (LVEF) by LV strain patterns based on cardiac magnetic resonance feature tracking (CMR-FT) and to explore the relationships between LV strain patterns and cardiac biomarkers in these patients, such as cardiac troponin (cTnT) and N-terminal prohormone of the brain natriuretic peptide (NT-proBNP).

**Methods:**

A total of 64 HCM patients with preserved LVEF and 33 healthy people were included in this study. All subjects underwent contrast-enhanced CMR, and all patients took blood tests for cTnT and NT-proBNP during hospitalization.

**Results:**

Despite the absence of a significant difference in LVEF between HCM patients and healthy controls, almost all global and segmental strains in radial, circumferential, and longitudinal directions in the HCM group deteriorated significantly as compared to controls (*p* < 0.05). Moreover, some global and segmental strains correlated significantly with NT-proBNP and cTnT in HCM patients, and the best correlations were global radial strain (GRS) (r = −0.553, *p* < 0.001) and mid-ventricular radial strain (MRS) (r = −0.582, *p* < 0.001), respectively, with a moderate correlation. The receiver operating characteristic (ROC) results showed that among the LV deformation parameters, GRS [area under the curve (AUC), 0.76; sensitivity, 0.49; specificity, 1.00], MRS (AUC, 0.81; sensitivity, 0.77; specificity, 0.79) demonstrated greater diagnostic accuracy to predict elevated NT-proBNP, and abnormal cTnT, respectively. Their cut-off values were 21.17 and 20.94%, respectively. Finally, all global strains demonstrated moderate, good, and excellent intra- and inter-observer reproducibility.

**Conclusion:**

LV strain patterns can be used to assess the subclinical cardiac function of HCM patients on the merit of being more sensitive than LVEF. In addition, LV strain patterns can detect serious HCM patients and may be helpful to non-invasively predict elevated NT-proBNP and cTnT.

## Introduction

As a commonly inherited cardiomyopathy with an incidence of 1:500, hypertrophic cardiomyopathy (HCM) is the most common cause of sudden death among young people (1). Its clinical diagnosis depends on LV hypertrophy, which cannot be accounted for by other reasons and is identified by cardiac magnetic resonance (CMR) or echocardiography (2). Left ventricular ejection fraction (LVEF) is usually regarded as the most frequently used index in assessing cardiac function and predicting the prognosis of HCM patients (3). However, as we know, LVEF in many HCM patients is normal (LVEF ≥ 50%). Given this condition, it seems unsuitable to depend only on LVEF to assess the cardiac function of HCM patients. Cardiac magnetic resonance feature tracking (CMR-FT), a post-processing technology with high sensitivity and good repeatability, can detect functional impairment of the heart before LVEF decreases and has thus been studied in many heart diseases (4, 5). Studies of HCM patients have shown that CMR-FT-based global strains in the radial, circumferential, and longitudinal directions decreased significantly compared to healthy controls, which can also be used to predict the prognosis of HCM patients (6–8). As two biomarkers of cardiac injuries exist in the blood circulation system, cardiac troponin (cTnT) and N-terminal prohormone of the brain natriuretic peptide (NT-proBNP) are used for many cardiac diseases (9–11). However, there are few studies reporting the relationships between left ventricular (LV) strain patterns and cardiac biomarkers in HCM patients with preserved LVEF. Given this gap, this present study is intended to use LV strain patterns to assess HCM patients with preserved LVEF and to explore the relationships between LV strain patterns and NT-proBNP (or cTnT) in HCM patients with preserved LVEF.

## Methods

### Patients and controls

A total of 64 HCM patients with preserved LVEF and 33 healthy people were included in this retrospective study at West China Hospital, Sichuan University, Chengdu, China. They all underwent contrast-enhanced CMR exams during their hospitalization from February 2018 to February 2022. HCM patients also took the biochemical tests on NT-proBNP and cTnT not exceeding ± 3 days before or after the CMR exams. During the same period, healthy subjects received contrast-enhanced CMR exams at West China Hospital, Sichuan University, Chengdu. According to the guidelines of the European Society of Cardiology (ESC) (12), HCM is defined as the maximum diastolic wall above 15 mm in thickness. The exclusion criteria for HCM patients are as follows: (1) age < 18; (2) LVEF ≤ 50%; (3) blood pressure ≥ 160/100 mmHg, complete bundle branch block, coronary stenosis higher than 20%, permanent mechanical device implant, myocardial resection, alcohol septal ablation or heart transplant, related valvular dysfunction, glomerular filtration rate (GFR) < 60 mL/min/1.73m^2^, malignant tumor, severe infection, connective tissue disease, and other systemic diseases. (4) suffering from diseases that cause elevated levels of adrenaline, glucocorticoids, and thyroxine—such as pheochromocytoma, Cushing's syndrome, primary thyroid hyperthyroidism, and sub-acute thyroiditis. (5) poor CMR image quality for post-processing. The threshold value of cTnT is 14 pg/mL (13). According to the 2017 American Heart Association (AHA) criteria, the cut-off value of NT-proBNP is 800 pg/mL (14). The criteria for excluding the controls included chronic disease, cardiovascular disease, family history, hypertension (blood pressure ≥ 140/90 mmHg), or severe arrhythmia. The Ethics Committee approved all data in this study of West China Hospital, Sichuan University, Chengdu, China, in agreement with the Helsinki Declaration.

### CMR protocol

CMR images were obtained using Siemens 3.0 T CMR scanners (Skyra; Siemens Medical Solutions, Erlangen, Germany) in a supine position with the head first. Electrocardiographic gating and respiratory gating were adopted during the scanning. Cine-CMR views, including 2- and 4-chamber long-axis views and a set of short-axis views covering the entire LV, were required using the sequence of balanced steady-state free-precession. Scan parameters included the field of view = 350–400 mm, repetition time/echo time = 3.0–3.6/1.5–1.8 ms, flip angle = 60°, and slice thickness = 8 mm. Late gadolinium enhancement (LGE) images were obtained 10 to 15 min after intravenous administration of gadodiamide (Magnevist; Bayer Schering Pharma, Berlin, Germany) at a speed of 0.2 mmol/kg scanned by the inversion recovery echo sequence. Its parameters included field of view = 350–400 mm, repetition time/echo time = 4.5–4.6/1.3–1.5 ms, flip angle =15°, inversion time = 200–300 ms, and slice thickness = 8 mm.

### CMR data analysis

**Figure 1:**
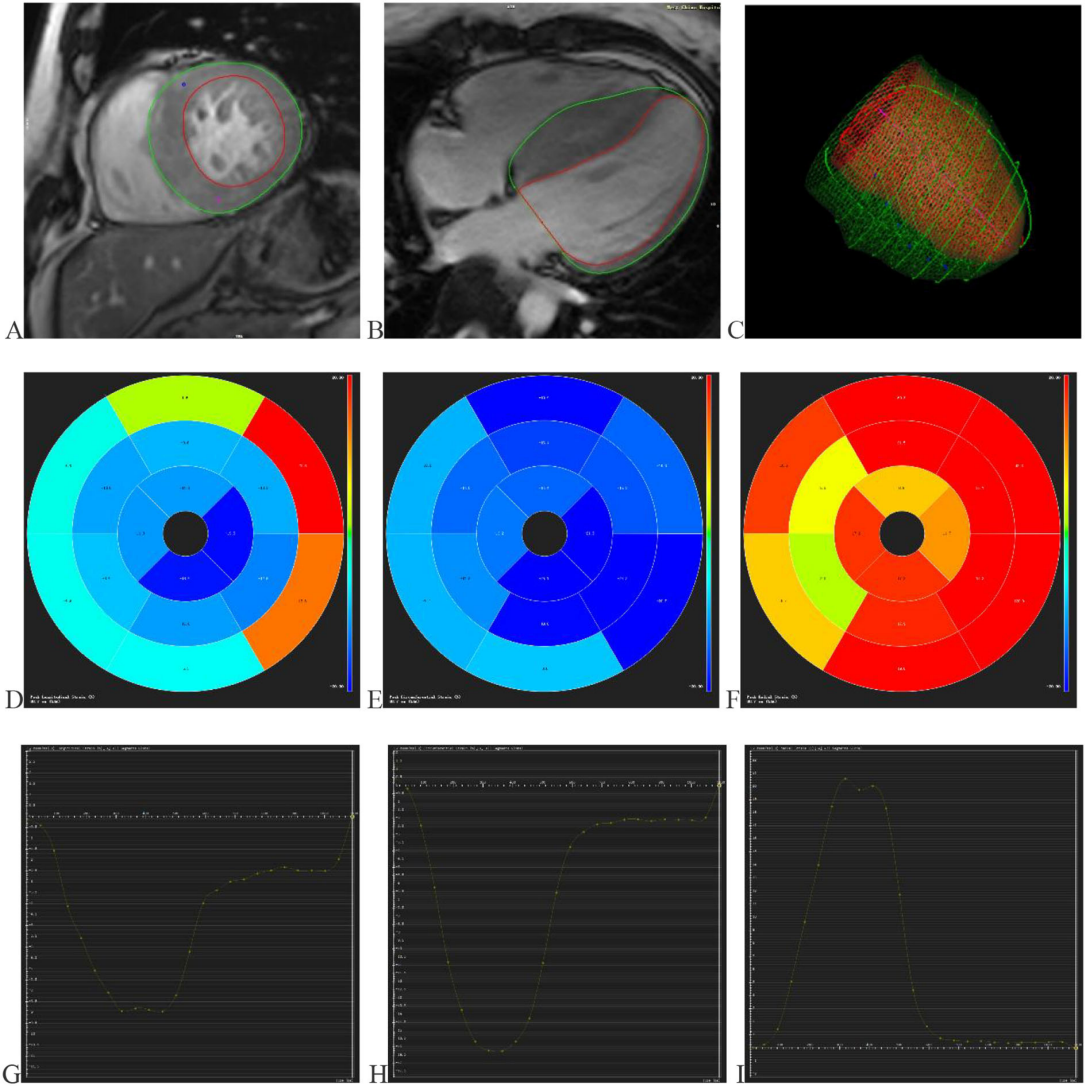
Displays the progress and outcomes of LV strains. **(A)** Delineating the LV endocardium and epicardium in the short-axis view. **(B)** Delineating the LV endocardium and epicardium in the 4-chamber long axis view. **(C)** The optimal endocardium and epicardium are shown in 3D space. The segmental strain round cake is mapped into 16 sections in the circumferential **(D)**, longitudinal **(E)**, and radial **(F)** directions. The global circumferential **(G)**, longitudinal **(H)**, and radial **(I)** strain curves in one cardiac cycle. LV (left ventricular).

Two radiologists conducted CMR data analysis with over 5 years of experience in imaging analysis. When they differed in opinion, they would negotiate with each other to achieve a consensus. The CMR image data acquired from the scanning workstation were loaded in software named “CVI.42” (Circle Cardiovascular Imaging, version 5.11, Calgary, AB, Canada) for analyzing LV function and characterization. First, a short-axis stock was dragged into the SHORT 3D module by drawing up the optimal endocardium and epicardium to acquire the basic cardiac function parameters, including LVEF, end-diastolic volume, end-systolic volume, and stroke volume. Meanwhile, a cardiac cycle measured maximum left ventricle wall thickness (MLVWT) during the end of diastole. Second, 2- and 4-chamber long-axis views and a set of short-axis views were transmitted into the Feature Tracking module. The optimal endocardium and epicardium were drawn up, excluding papillary muscles (Figure 1). Then CVI.42 would automatically calculate the cardiac strain parameters such as global and segmental strains (Figure 1). LV contractility was evaluated by global radial strain (GRS), global circumferential strain (GCS), global longitudinal strain (GLS), apical radial strain (ARS), apical circumferential strain (ACS), apical longitudinal strain (ALS), mid-ventricular radial strain (MRS), mid-ventricular circumferential strain (MCS), mid-ventricular longitudinal strain (MLS), basal radial strain (BRS), basal circumferential strain (BCS), and basal longitudinal strain (BLS).

### Repeatability

Scans of 20 randomly selected subjects in patients and controls were repeatedly examined by the same observer with an interval of 2 weeks to represent the intra-observer repeatability by calculating the intra-class correlation coefficient (ICC). ICC also represented inter-observer repeatability, which was generated by measuring the same subject by two independent, experienced, and double-blinded observers.

### Statistical analysis

Statistical analysis was performed by IBM SPSS (V. 26.0, ARMONK, NY, USA). The mean ± standard deviation (SD) or median with interquartile range (IQR) [25%, 75%] was used to represent continuous variables. The Chi-square test or Fisher's exact test was performed to assess the difference in classified data such as sex, clinical symptoms, etc. The Mann–Whitney U-test or Student's *t*-test was used to compare the continuous variables, including MLVWT, LVEF, strain, etc. The difference in New York Heart Association (NYHA) classification was presented by the Kruskal–Wallis rank test. The receiver operating characteristic (ROC) curve was analyzed to assess the predictive value of LV strain parameters for elevated NT-proBNP and abnormal cTnT in HCM patients. ICC was used to assess the intra- and inter-observer consistency, whose values were defined as the 95% confidence interval (CI) lower limit exhibited poor (< 0.5), moderate (0.5–0.75), good (0.75–0.9), or excellent (> 0.9) reliability (15). The Spearman's rank correlation coefficient was calculated to assess the correlations between continuous parameters when a two-tailed *p* < 0.05, and the difference in the corresponding data was considered statistically significant.

## Results

### Patient characteristics

There were 64 patients and 33 healthy controls included in the study. There was no significant difference in age (mean age, 48 ± 10 years vs. 50 ± 14 years; *p* = 0.365), LVEF (61.02 ± 5.40 % vs. 62.92 ± 8.00 %; *p* = 0.170) and sex (39 men and 25 women vs. 20 men and 13 women; *p* = 0.975) between the two groups. It can be seen that, compared to controls, the MLVWT in HCM patients significantly thickened. In the HCM group, there were 53 (82.81%) patients showing positive LGE, 7 (10.94%) showing atrial fibrillation, 42 (65.63%) showing left ventricular outflow tract obstruction (LVOTO), and 27 (42.19%) showing mitral regurgitation. According to NYHA classification, 13 (20.31%) individuals were NYHA class I, 40 (62.50%) were NYHA class II, 8 (12.50%) were NYHA class III, and 3 (4.69%) were NYHA class IV in this group. Interestingly, almost all LGE positive parts overlapped with the regions of myocardial hypertrophy and were mainly in the mid-wall. Other basic information is supplemented in Table 1.

**Table 1:** Basic information about HCM patients and healthy controls.

**Parameter, unit**	**Controls**	**HCM**	***p-*** **value**
	**(*n* = 33)**	**(*n* = 64)**	
Age, years, (SD)	48 (10)	50 (14)	0.365
Male, *n (%)*	20 (73.33)	39 (60.94)	0.975
Heart rate, bpm, (SD)	N/A	72 (11)	
Respiration, rpm, (SD)	N/A	20 (1)	
Height, cm, (SD)	N/A	162 (9)^a^	
Weight, kg, (SD)	N/A	62.6 (10.5)	
Dizzy, *n (%)*	N/A	10 (15.63)	-
Bosom frowsty, *n (%)*	N/A	30 (46.88)	-
Bosom painful, *n (%)*	N/A	29 (45.31)	-
Palpitation, *n (%)*	N/A	15 (23.44)	-
Amaurosis, *n (%)*	N/A	11 (17.19)	-
Syncope, *n (%)*	N/A	15 (23.44)	-
Family histories, *n (%)*	N/A	3 (4.69)	-
Personal histories			
Smoking, *n (%)*	N/A	21 (32.81)	-
Drinking, *n (%)*	N/A	9 (14.06)	-
Atrial fibrillation, *n (%)*	0	7 (10.94)	0.144
NYHA classification (I/II/III/IV)	N/A	13/40/8/4	-
Volumes and functions			
LVEF, % (SD)	61.02 (5.40)	62.92 (8.00)	0.170
LVEDV, mL (SD)	127.54 (27.81)	133.57 (29.59)	0.334
LVESV, mL (SD)	50.00 (14.26)	50.64 (17.41)	0.856
LVSV, mL (SD)	77.53 (16.59)	82.77 (17.69)	0.161
MLVWT, mm, (SD)	8.61 (1.69)	22.00 (5.53)	<0.001^*^
RVEF, % (SD)	56.60 (7.35)	58.53 (9.33)	0.268
RVEDV, mL (SD)	119.12 (29.37)	95.15 (25.74)	<0.001^*^
RVESV, mL (IQR)	49.40 (40.10, 63.90)	39.00 (31.40, 49.58)	0.004^*^
RVSV, mL (SD)	66.31 (14.11)	55.48 (16.60)	0.002^*^
LGE presence, *n (%)*	0	53 (82.81)	<0.001^*^
Mitral regurgitation, *n (%)*	0	27 (42.19)	<0.001^*^
LVOTO, *n (%)*	0	42 (65.62)	<0.001^*^
Biomarkers, pg/mL			
NT-proBNP (IQR)	N/A	989.00 (356.50, 1869.75)^b^	-
cTnT (IQR)	N/A	14.25 (9.98, 22.83)	-

Statistical significance was defined as p < 0.05. Data were expressed as mean (SD), absolute numbers (percentages), or median (IQR 25%, 75%).

* p < 0.05 vs. controls. HCM, hypertrophic cardiomyopathy; SD, standard deviation; IQR, interquartile range; NYHA, New York Heart Association; LVEF, left ventricular ejection fraction; LVEDV, left ventricular end-diastolic volume; LVESV, left ventricular end-systolic volume; LVSV, left ventricular stroke volume; RVEF, right ventricular ejection fraction; RVEDV, right ventricular end-diastolic volume; RVESV, right ventricular end-systolic volume; RVSV, right ventricular stroke volume; LGE, late gadolinium enhancement; LVOTO, left ventricular outflow tract obstruction; NT-proBNP, N-terminal prohormone of the brain natriuretic peptide; and cTnT, cardiac troponin T.

a Height was available in n = 61. b NT-proBNP was available in n = 62.

### LV myocardial deformation in HCM patients and healthy controls

Even though no significant difference was observed in LVEF (61.02 ± 5.40 % vs. 62.92 ± 8.00 %; *p* = 0.170) between HCM patients and controls, compared controls, all LV global and segmental strains in HCM patients showed a trend of impairment with statistical significance except for ARS and BCS. Those above specific values can be seen in Table 2.

**Table 2:** LV strains in HCM patients and healthy controls.

**Strain, %**	**Controls**	**HCM**	***p–*** **value**
	**(*n* = 33)**	**(*n* = 64)**	
GRS (IQR)	36.84 (27.94, 41.29)	24.60 (20.69, 35.09)	0.001^*^
GCS (IQR)	−21.01 (−22.84, −17.94)	−17.79 (−20.92, −12.14)	0.007^*^
GLS (SD)	−14.67 (3.14)	−9.44 (3.41)	<0.001^*^
ARS (IQR)	29.53 (23.83, 43.73)	26.36 (19.96, 38.09)	0.201
ACS (IQR)	−23.91 (−26.82, −18.63)	−19.68 (−24.16, −16.11)	0.016^*^
ALS (SD)	−17.85 (2.69)	−14.00 (3.73)	<0.001^*^
MRS (IQR)	28.62 (24.09, 38.51)	20.73 (15.01, 29.73)	<0.001^*^
MCS (SD)	−20.50 (3.95)	−17.18 (4.35)	<0.001^*^
MLS (SD)	−14.57 (3.51)	−8.64 (4.59)	<0.001^*^
BRS (IQR)	46.35 (36.87, 59.07)	34.31 (27.71, 41.73)	<0.001^*^
BCS (IQR)	−18.29 (−21.19, −16.11)	−16.88 (−19.28, −14.66)	0.114
BLS (IQR)	−10.99 (−14.46, −7.61)	−5.32 (−9.95, 5.07)	<0.001^*^

Statistical significance was defined as p < 0.05. Data were expressed as mean (SD) or median (IQR 25%, 75%).

* p < 0.05 vs. controls. LV left ventricular; HCM, hypertrophic cardiomyopathy; SD, standard deviation; IQR, interquartile range; GRS, global radial strain; GCS, global circumferential strain; GLS, global longitudinal strain; ARS, apical radial strain; ACS, apical circumferential strain; ALS, apical longitudinal strain; MRS, mid–ventricular radial strain; MCS, mid–ventricular circumferential strain; MLS, mid–ventricular longitudinal strain; BRS, basal radial strain; BCS, basal circumferential strain; and BLS, basal longitudinal strain.

### NT-ProBNP

**Figure 2:**
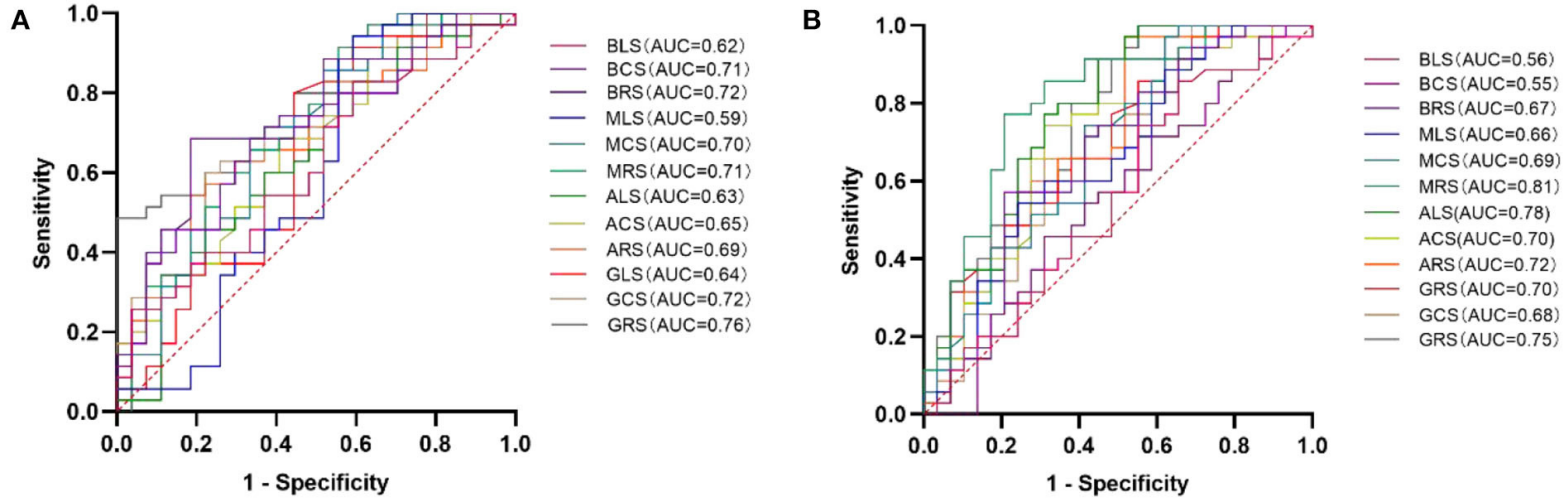
ROC curve analysis of LV deformation parameters for predicting elevated NT-proBNP **(A)** and cTnT **(B)** in HCM patients. HCM, hypertrophic cardiomyopathy; LV, left ventricular; ROC, receiver operating characteristic; AUC, area under the curve; GRS, global radial strain; GCS, global circumferential strain; GLS, global longitudinal strain; ARS, apical radial strain; ACS, apical circumferential strain; ALS, apical longitudinal strain; MRS, mid-ventricular radial strain; MCS, mid-ventricular circumferential strain; MLS, mid-ventricular longitudinal strain; BRS, basal radial strain; BCS, basal circumferential strain; BLS, basal longitudinal strain; NT-proBNP, N-terminal prohormone of the brain natriuretic peptide; and cTnT, cardiac troponin T.

There were 35 (56.45%) HCM patients in the elevated NT-proBNP group (≥ 800 pg/mL) and 27 (43.55%) HCM patients in the non-elevated NT-proBNP group (< 800 pg/mL). However, the indicators of two patients were unfortunately lost. Between the two groups, there was no significant difference in age (mean age, 49 ± 13 years vs. 50 ± 16 years; *p* = 0.765) and LVEF (63.97 ± 7.23% vs. 62.06 ± 8.43 %; *p* = 0.351). However, compared to the non-elevated NT-proBNP group, GRS, GCS, ARS, ACS, MRS, MCS, BRS, and BCS decreased significantly in the elevated NT-proBNP group (*p* < 0.05). Although the differences in GLS, ALS, MLS, and BLS between the two groups were not statistically significant (*p* > 0.05), they still exhibited a declining trend. The accurate values of all the above strains across the two groups are presented in Table 3. Among the LV deformation parameters in HCM patients, GRS (r = −0.553, *p* < 0.001) showed the best correlation with NT-proBNP level in the global strains, while MRS (r = −0.475, *p* < 0.001) showed the best correlation with NT-proBNP level in the segmental strains (Table 4). In addition, GRS showed the highest predictive value for elevated NT-proBNP [area under the curve (AUC), 0.76; sensitivity, 0.49; specificity, 1.00] in the global strains, while BRS showed the highest predictive value for elevated NT-proBNP (AUC, 0.72; sensitivity, 0.69; specificity, 0.82) in the segmental strains (Figure 2). Their cut-off values were 21.17 and 33.57%, respectively.

**Table 3:** LV strains in HCM patients with and without elevated NT–proBNP.

**Strain, %**	**NT–proBNP < 800 pg/mL**	**NT–proBNP** **≥800 pg/mL**	***p–*** **value**
	**(*n* = 27)**	**(*n* = 35)**	
GRS (IQR)	30.00 (23.96, 41.42)	21.87 (18.68, 28.61)	0.001^*^
GCS (SD)	−19.63 (4.02)	−16.41 (3.62)	0.002^*^
GLS (IQR)	−11.35 (−12.90, −8.29)	−9.37 (−10.57, −6.11)	0.058
ARS (IQR)	31.08 (25.89, 40.98)	22.54 (17.26, 32.33)	0.011^*^
ACS (SD)	−21.62 (3.45)	−18.45 (5.41)	0.030^*^
ALS (SD)	−14.79 (3.44)	−13.35 (3.17)	0.093
MRS (SD)	28.12 (12.93)	19.16 (7.90)	0.003^*^
MCS (SD)	−18.90 (4.51)	−15.71 (3.66)	0.003^*^
MLS (IQR)	−9.38 (−13.54, −4.68)	−9.40 (−11.20, 6.47)	0.230
BRS (IQR)	38.34 (34.13, 53.56)	30.17 (24.59, 38.40)	0.003^*^
BCS (IQR)	−18.45 (−19.55, −16.20)	−16.61 (−18.46, −14.08)	0.005^*^
BLS (IQR)	−5.63 (−10.75, −2.27)	−3.90 (−8.45, 6.66)	0.109

Statistical significance was defined as p < 0.05. Data were expressed as mean (SD) or median (IQR 25%, 75%).

* p < 0.05 vs. controls. LV left ventricular; HCM, hypertrophic cardiomyopathy; SD, standard deviation; IQR, interquartile range; GRS, global radial strain; GCS, global circumferential strain; GLS, global longitudinal strain; ARS, apical radial strain; ACS, apical circumferential strain; ALS, apical longitudinal strain; MRS, mid–ventricular radial strain; MCS, mid–ventricular circumferential strain; MLS, mid–ventricular longitudinal strain; BRS, basal radial strain; BCS, basal circumferential strain; BLS, basal longitudinal strain; and NT–proBNP, N–terminal prohormone of the brain natriuretic peptide.

**Table 4:** Correlations of LV strains with NT–proBNP in HCM patients.

**Strain (%)**	**NT–proBNP (pg/mL)**	
	**Rho**	***p***
GRS	−0.553	<0.001
GCS	0.428	0.001
GLS	0.281	0.027
ARS	−0.381	0.002
ACS	0.281	0.027
ALS	0.315	0.013
MRS	−0.475	<0.001
MCS	0.434	<0.001
MLS	0.225	0.079
BRS	−0.430	<0.001
BCS	0.335	0.008
BLS	0.126	0.327

Statistical significance was defined as p < 0.05. LV left ventricular; HCM, hypertrophic cardiomyopathy; GRS, global radial strain; GCS, global circumferential strain; GLS, global longitudinal strain; ARS, apical radial strain; ACS, apical circumferential strain; ALS, apical longitudinal strain; MRS, mid–ventricular radial strain; MCS, mid–ventricular circumferential strain; MLS, mid–ventricular longitudinal strain; BRS, basal radial strain; BCS, basal circumferential strain; BLS, basal longitudinal strain; and NT–proBNP, N–terminal prohormone of the brain natriuretic peptide.

### cTnT

There were 29 (45.31%) patients in the normal cTnT group (< 14 pg/mL) and 35 (54.69%) patients in the abnormal cTnT group (≥ 14 pg/mL). There was no significant difference between the two groups in age (mean age, 53 ± 12 years vs. 48 ± 16 years; *p* = 0.243) and sex (16 men and 13 women vs. 23 men and 12 women; *p* = 0.390). Compared to that in the normal cTnT group, GRS, GCS, GLS, ARS, ACS, ALS, MRS, MCS, and BRS in the abnormal cTnT group decreased significantly (*p* < 0.05), and MLS, BCS, and BLS also tended to decline, regardless of hardly any statistical difference (*p* > 0.05). These detailed data are presented in Table 5. Among the LV deformation parameters in HCM patients, GRS (r = −0.535, *p* < 0.001) showed the best correlation with cTnT level in the global strains, while MRS (r = −0.582, *p* < 0.001) showed the best correlation with cTnT level in the segmental strains (Table 6). Furthermore, GRS showed the highest predictive value for abnormal cTnT (AUC, 0.75; sensitivity, 1.00; specificity, 0.45) in the global strains, while MRS showed the highest predictive value for abnormal cTnT (AUC, 0.81; sensitivity, 0.77; specificity, 0.79) in the segmental strains (Figure 2). Their cut-off values were 37.91 and 20.94%, respectively.

**Table 5:** LV strains in HCM patients with and without elevated cTnT.

**Strain, %**	**cTnT < 14 pg/mL**	**cTnT ≥14 pg/mL**	***p–*** **value**
	**(*n* = 29)**	**(*n* = 35)**	
GRS (SD)	35.00 (14.18)	23.50 (6.26)	<0.001^*^
GCS (SD)	−19.24 (4.54)	−16.73 (3.35)	0.014^*^
GLS (SD)	−10.75 (3.33)	−8.36 (3.12)	0.004^*^
ARS (IQR)	30.17 (24.22, 53.42)	24.45 (17.60, 32.33)	0.003^*^
ACS (SD)	−22.11 (5.81)	−18.17 (5.15)	0.006^*^
ALS (SD)	−15.76 (3.26)	−12.54 (2.74)	<0.001^*^
MRS (SD)	29.59 (12.06)	18.06 (7.34)	<0.001^*^
MCS (SD)	−18.85 (4.63)	−15.80 (3.62)	0.004^*^
MLS (SD)	−9.80 (4.77)	−7.67 (4.27)	0.065
BRS (IQR)	38.34 (32.88, 55.44)	30.17 (26.56, 38.78)	0.022^*^
BCS (SD)	−17.23 (4.41)	−17.23 (2.63)	0.963
BLS (IQR)	−5.56 (−10.71, 4.59)	−5.31 (−8.88, 5.58)	0.434

Statistical significance was defined as p < 0.05. Data were expressed as mean (SD) or median (IQR 25%, 75%).

* p < 0.05 vs. controls. LV left ventricular; HCM, hypertrophic cardiomyopathy; SD, standard deviation; IQR, interquartile range; GRS, global radial strain; GCS, global circumferential strain; GLS, global longitudinal strain; ARS, apical radial strain; ACS, apical circumferential strain; ALS, apical longitudinal strain; MRS, mid–ventricular radial strain; MCS, mid–ventricular circumferential strain; MLS, mid–ventricular longitudinal strain; BRS, basal radial strain; BCS, basal circumferential strain; BLS, basal longitudinal strain; and cTnT, cardiac troponin.

**Table 6:** Correlations of LV strains with cTnT in HCM patients.

**Strain (%)**	**cTnT (pg/mL)**	
	**Rho**	***P***
GRS	−0.535	<0.001
GCS	0.401	0.001
GLS	0.408	0.001
ARS	−0.455	<0.001
ACS	0.398	0.001
ALS	0.537	<0.001
MRS	−0.582	<0.001
MCS	0.411	0.001
MLS	0.330	0.008
BRS	−0.343	0.005
BCS	0.088	0.488
BLS	0.126	0.321

Statistical significance was defined as p < 0.05. LV left ventricular; HCM, hypertrophic cardiomyopathy; GRS, global radial strain; GCS, global circumferential strain; GLS, global longitudinal strain; ARS, apical radial strain; ACS, apical circumferential strain; ALS, apical longitudinal strain; MRS, mid–ventricular radial strain; MCS, mid–ventricular circumferential strain; MLS, mid–ventricular longitudinal strain; BRS, basal radial strain; BCS, basal circumferential strain; BLS, basal longitudinal strain; and cTnT, cardiac troponin T.

### Reproducibility

GLS exhibited a good intra- (ICC = 0.94; 95% CI, 0.86–0.97) and an excellent inter-observer (ICC = 0.97; 95% CI, 0.92–0.99) reproducibility. GRS also exhibited a good intra- (ICC = 0.93; 95% CI, 0.83–0.97) and a moderate inter-observer (ICC = 0.85; 95% CI, 0.65–0.94) reproducibility, whereas GCS exhibited a moderate intra- (ICC = 0.85; 95% CI, 0.66–0.94) and an excellent inter-observer (ICC = 0.98; 95% CI, 0.94–0.99) reproducibility.

## Discussion

This study has made the following findings. First, despite the insignificant difference in LVEF between controls and HCM patients, all LV global and segmental strains in HCM patients exhibited a declining trend with statistical significance except for ARS and BCS. Second, NT-proBNP levels in most HCM patients increased. Compared to the non-elevated NT-proBNP group (< 800 pg/mL), some global and segmental strains, including GRS, GCS, ARS, ACS, MRS, MCS, BRS, and BCS in the elevated NT-proBNP group, decreased significantly. Third, the cTnT level in some HCM patients increased. In addition, LV global and segmental strains in the abnormal cTnT group, except for MLS, BCS, and BLS, had decreased significantly compared to the normal cTnT group in HCM patients. Fourth, some strains were correlated considerably with NT-proBNP and cTnT in HCM patients, with the optimal correlations falling on GRS in the global strains and MRS in the segmental strains.

Furthermore, BRS and MRS showed the highest predictive value for elevated NT-proBNP and abnormal cTnT in the segmental strains, respectively. Among the global strains, GRS showed the highest predictive value for elevated NT-proBNP and abnormal cTnT. Lastly, global strains in the three directions presented moderate to slightly high intra- and inter-observer reproducibility. The best intra-and inter-observer reproducibility was exhibited by GLS and GCS, respectively.

### LV myocardial deformation

First, this study focused on HCM with preserved LVEF in hospitalized patients. Therefore, the percentage of HCM patients with LGE (82.81%) and LVOTO (65.63%) was high compared with a prior study that focused on all HCM patients with preserved LVEF (16). Such an approach helped us learn more accurately about more serious HCM patients of this subtype, thus promoting their clinical management. Second, consistent with a previous study (6), we also found that even though the difference in LVEF between the two groups was not significant, LV strains of HCM patients were impaired significantly compared with healthy people, which may be related to the fact that, in the progress of HCM, cardiac hypertrophy and coronary microvascular dysfunction result in ischemia and fibrosis, which lead to a decrease in myocardial contraction ability (17–19). Therefore, CMR-FT can indicate changes in subclinical myocardial contractility in HCM patients independent of LVEF and heart failure (HF) classification. Besides, compared with using LVEF for assessing global cardiac function, CMR-FT can more clearly and accurately demonstrate the impaired local cardiac function by means of changes in segmental strains.

Furthermore, we found that global strains from the three directions showed moderate to better intra- and inter-observer reproducibility. GLS showed the best intra-observer reproducibility, and GLS showed the best inter-observer reproducibility. Some authors proposed that the poor reproducibility for GRS might be due to the geometry of the heart with analysis in a plane of movement with the smallest potential diameter for tracking (20), while other people speculated that the lower reproducibility of GRS might be correlated with the measurement of the interaction of the epicardium and endocardium during the tracking, which is not necessary for the derivation of GLS and GCS (21).

### NT-ProBNP and cTnT

NT-proBNP, a neurohormone, is synthesized and released by atrial cells in normal organs. Nonetheless, with the development of HF, ventricular cells can also generate it in response to wall stress (22). NT-proBNP has emerged as a useful and reliable biomarker in diagnosing and predicting HF (23). Furthermore, it may also be conducive to the risk stratification of some cardiac diseases (24, 25). Compared with BNP, NT-proBNP is more stable on the merit of its longer half-life (23). Some studies reported that NT-proBNP was associated with cardiac fibrosis, deterioration of cardiac function, and prognosis in HCM patients (26–29). According to the 2016 ESC guideline (30), the NT-proBNP cutoff value is 125 pg/mL, whereas the 2017 AHA criteria suggested using a threshold of 800 pg/mL (14). A study indicated that compared to the cut-off value of NT-proBNP of the ESC guideline, the AHA criteria are more conducive to risk stratification in HCM patients (31). Accordingly, in this study, we chose 800 pg/mL as the threshold criterion for the group. c-TnT was considered a preferred biomarker in detecting cardiac injury owing to its high sensitivity and specificity (32). Some studies found that as cTnT could be elevated in many HCM patients, it could predict the prognosis and help with risk stratification in HCM patients (32–35). In this study, we found that there were 35 patients (56.45%) with elevated NT-proBNP and 35 (54.69%) patients in the abnormal cTnT group, which is consistent with the previous studies (28, 29, 34, 36). Furthermore, we also found that some global and segmental strains decreased significantly in the elevated NT-proBNP group compared to the non-elevated NT-proBNP group. The performance of the groups with and without elevated cTnT was similar to that of the NT-proBNP group. Additionally, some strains were significantly correlated with NT-proBNP and cTnT in HCM patients. The best strains were GRS (NT-proBNP: r = −0.553, *p* < 0.001; cTnT: r = −0.535, *p* < 0.001) in the global strains and MRS (NT-proBNP: r = −0.475, *p* < 0.001; cTnT: r = −0.582, *p* < 0.001) in the segmental strains with moderate correlations. According to ROC analysis, we found that BRS (AUC, 0.72; sensitivity, 0.69; specificity, 0.82) and MRS (AUC, 0.81; sensitivity, 0.77; specificity, 0.79) showed the highest predictive value for elevated NT-proBNP and abnormal cTnT in the segmental strains, respectively. Their cut-off values were 33.57 and 20.94%, respectively. Among the global strains, GRS showed the highest predictive value for elevated NT-proBNP (AUC, 0.76; sensitivity, 0.49; specificity, 1.00) and abnormal cTnT (AUC, 0.75; sensitivity, 1.00; specificity, 0.45). Their cut-off values were 21.17 and 37.91%, respectively. Therefore, LV strain patterns can be used to monitor changes in HCM and detect more severe cases to improve the health management of HCM patients and may be used to non-invasively predicate elevated NT-proBNP and cTnT to avoid the risks caused by clinical operations. What's more, some studies have indicated that CMR-FT can be used to predict the adverse prognosis of HCM patients (6–8). Certainly, it warrants to be verified in future studies as to whether predicting the prognosis of HCM patients by using CMR-FT together with cardiac biomarkers simultaneously is better than such prediction by using them separately.

## Limitation

First, as this is a single-centered retrospective study based upon a small sample focusing on inpatients with HCM, the applicable range of its conclusion cannot be extended to all HCM patients with preserved LVEF. Moreover, some multi-centered and extensive sample prospective studies are needed to validate the accuracy and significance of the views proposed by this study. Second, as a retrospective study, some data were missing, such as some values of NT-proBNP and height. Moreover, as this is a retrospective study, it is hardly possible to explore the relationship between the strain and other parameters based on CMR, such as T_1_ mapping, extracellular volume, and T_2_ mapping. Meanwhile, the comparison between CMR-FT and other imaging techniques that can assess cardiac strain, such as speckle tracking echocardiography, was also impossible. Finally, this study focused on the correlations between LV strain patterns and cardiac biomarkers in HCM patients. However, as mentioned in the discussion section, the ability to predict the prognosis of HCM patients by combining CMR-FT and cardiac biomarkers is worth further study.

## Conclusion

LV strain patterns can be used to assess the subclinical cardiac function of HCM patients on the merit of being more sensitive than LVEF. In addition, LV strain patterns can detect serious HCM patients and may be helpful to non-invasively predict elevated NT-proBNP and cTnT.

## Data availability statement

The original contributions presented in the study are included in the article/supplementary material, further inquiries can be directed to the corresponding author.

## Ethics statement

The studies involving human participants were reviewed and approved by Ethics Committee of Sichuan University West China Hospital. Written informed consent for participation was not required for this study in accordance with the national legislation and the institutional requirements.

## Author contributions

LZ and YW planned and wrote the manuscript. BH, LW, DZ, and FG edited the manuscript. All authors contributed to the article and approved the submitted version.

## Funding

The project was supported by the National Natural Science Foundation of China (Nos. 81930046 and 81829003).

## Conflict of interest

The authors declare that the research was conducted in the absence of any commercial or financial relationships that could be construed as a potential conflict of interest.

## Publisher's note

All claims expressed in this article are solely those of the authors and do not necessarily represent those of their affiliated organizations, or those of the publisher, the editors and the reviewers. Any product that may be evaluated in this article, or claim that may be made by its manufacturer, is not guaranteed or endorsed by the publisher.

## Abbreviations

HCM: Hypertrophic cardiomyopathy
CMR: Cardiac magnetic resonance
LVEF: Left ventricular ejection fraction
CMR-FT: Cardiac magnetic resonance feature tracking
NT-proBNP: N-terminal prohormone of the brain natriuretic peptide
cTnT: Cardiac troponin T
LV: Left ventricular
LGE: Late gadolinium enhancement
MLVWT: Maximum LV wall thickness
GRS: Global radial strain
GCS: Global circumferential strain
GLS: Global longitudinal strain
ARS: Apical radial strain
ACS: Apical circumferential strain
ALS: Apical longitudinal strain
MRS: Mid-ventricular radial strain
MCS: Mid-ventricular circumferential strain
MLS: Mid-ventricular longitudinal strain
BRS: Basal radial strain
BCS: Basal circumferential strain
BLS: Basal longitudinal strain
ICC: Intraclass correlation coefficient
SD: Standard deviation
IQR: Interquartile range
NYHA: New York Heart Association
ROC: Receiver operating characteristic
CI: Confidence interval
LVOTO: Left ventricular outflow tract obstruction
AUC: Area under the curve.
